# Trifluoroethanol Modulates Amyloid Formation by the All α-Helical URN1 FF Domain

**DOI:** 10.3390/ijms140917830

**Published:** 2013-08-30

**Authors:** Patrizia Marinelli, Virginia Castillo, Salvador Ventura

**Affiliations:** 1 Institute of Biotechnology and Biomedicine, Autonomous University of Barcelona, Bellaterra E-08193, Spain; E-Mails: patriziamrnll864@gmail.com (P.M.); virginia.castillo.cano@uab.cat (V.C.); 2 Department of Biochemistry and Molecular Biology, Autonomous University of Barcelona, Bellaterra E-08193, Spain

**Keywords:** α-helix, amyloid, FF domain, trifluoroethanol, molten globule

## Abstract

Amyloid fibril formation is implicated in different human diseases. The transition between native α-helices and nonnative intermolecular β-sheets has been suggested to be a trigger of fibrillation in different conformational diseases. The FF domain of the URN1 splicing factor (URN1-FF) is a small all-α protein that populates a molten globule (MG) at low pH. Despite the fact that this conformation maintains most of the domain native secondary structure, it progressively converts into β-sheet enriched and highly ordered amyloid fibrils. In this study, we investigated if 2,2,2-trifluoroethanol (TFE) induced conformational changes that affect URN1-FF amyloid formation. Despite TFE having been shown to induce or increase the aggregation of both globular and disordered proteins at moderate concentrations, we demonstrate here that in the case of URN1-FF it reinforces its intrinsic α-helical structure, which competes the formation of aggregated assemblies. In addition, we show that TFE induces conformational diversity in URN1-FF fibrils, in such a way that the fibrils formed in the presence and absence of the cosolvent represent different polymorphs. It is suggested that the effect of TFE on both the soluble and aggregated states of URN1-FF depends on its ability to facilitate hydrogen bonding.

## 1. Introduction

Protein misfolding and aggregation underlie an increasing number of human diseases that include highly debilitating disorders like Alzheimer’s and Parkinson’s disease, type II diabetes or even some types of cancer [1,2]. Despite the proteins being involved in these pathologies, they are not related in sequential or structural terms. In many cases, their aggregation involves the acquisition of a common cross-β conformation leading to the formation of amyloid fibrils [3]. Moreover, the adoption of such macromolecular structures is not restricted to disease-linked polypeptides and might constitute a generic feature of proteins chains [4,5], since the non-covalent interactions characteristic of compact native structures resemble those stabilizing amyloid conformations [6].

Biophysical characterization of protein aggregation usually exploit cosolvents to trigger changes in the protein conformation or environment that allows the study of both the conformers involved in the self-assembly reaction and its kinetics in a controlled manner [7,8]. The fluorinated alcohol 2,2,2-trifluoroethanol (TFE) is perhaps the most used cosolvent to investigate protein aggregation [9]. TFE promotes a gradual coil-to-helix transition in peptides and disordered proteins [10,11], which attain maximal ellipticity at ~30%–40% *v*/*v* TFE. At the same concentrations, TFE usually results in globular proteins denaturation, inducing the formation of non-native α-helices. The presence of TFE at lower concentrations, typically 10%–40%, *v*/*v* has been shown to induce the aggregation of different globular proteins [12–17].

In many occasions pathological protein aggregation and fibril formation result from the population of partially folded conformations [18–20]. In native structures, proteins locate most of their hydrophobic side-chains and main-chain hydrogen bond formers inaccessible to solvent, thus reducing anomalous intermolecular bonding and aggregation. However, strong denaturing conditions usually render unfolded, but soluble protein conformations because the non-covalent inter- and intramolecular contacts that a protein may form are impeded. This explains why conditions promoting the population of partially folded conformations become more favorable to aggregation than native or unfolded states. At mild concentrations, TFE is a cosolvent that diminishes native tertiary interactions and induces partly folded states of polypeptides by disrupting hydrophobic contacts without preventing establishment of intermolecular interactions. In this respect, a TFE promoted partly folded state and an aggregation-prone conformational state of a pathological protein are similar. Most of the proteins for which TFE-induced amyloid formation has been studied consist of at least one preformed β-sheet element in their native structure. Intermolecular docking between these destabilized and partially exposed secondary structures in TFE-promoted partially folded states is thought to trigger the initial self-assembly reaction of these polypeptides [13,16]. In contrast, little is known about the effect of TFE on the aggregation of all α-helical proteins, where preformed β-sheet conformations are not present in their aggregation-competent states.

**Figure 1:**
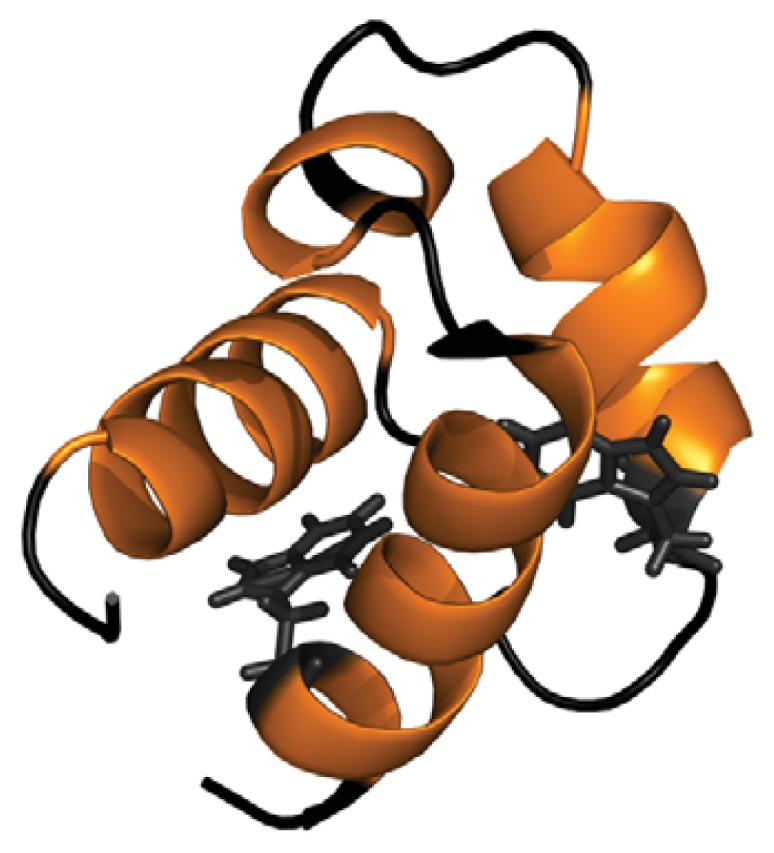
Ribbon representation of the structure of the URN1-FF domain. Tryptophan residues are shown in black. The Protein Data Bank accession code for the structure is 2JUC. This figure was prepared with PyMOL.

FF domains are small protein-protein interaction modules consisting of 50–70 residues often organized in tandem arrays, which display two conserved Phe residues at the *N*- and *C*-termini [21]. These domains consist of three α-helices arranged as an orthogonal bundle with a 3_10_ helix in the loop connecting the second and the third helix (Figure 1) [22–24]. They work in RNA splicing, signal transduction and transcription pathways and are present in different eukaryotic nuclear transcription and splicing factors. The conformational properties of this domain are receiving increasing attention, since its folding reaction involves the transient population of an on-pathway intermediate whose structure has been has been solved at atomic resolution by NMR, providing thus high-quality data on the structural constraints driving the early stages of folding [25,26]. In a recent work, we have shown that the yeast URN1-FF domain (Figure 1) populates a molten globule (MG) state under mild denaturing conditions at low pH, which, despite being devoid of any detectable β-sheet element, self-assembles into highly ordered amyloid fibrils [27]. Here, we investigate the impact of TFE-induced structural rearrangements on the process of amyloid formation by this all α-helical protein.

## 2. Results and Discussion

### 2.1. Conformational Properties of URN1-FF at Low pH in the Presence of TFE

**Figure 2:**
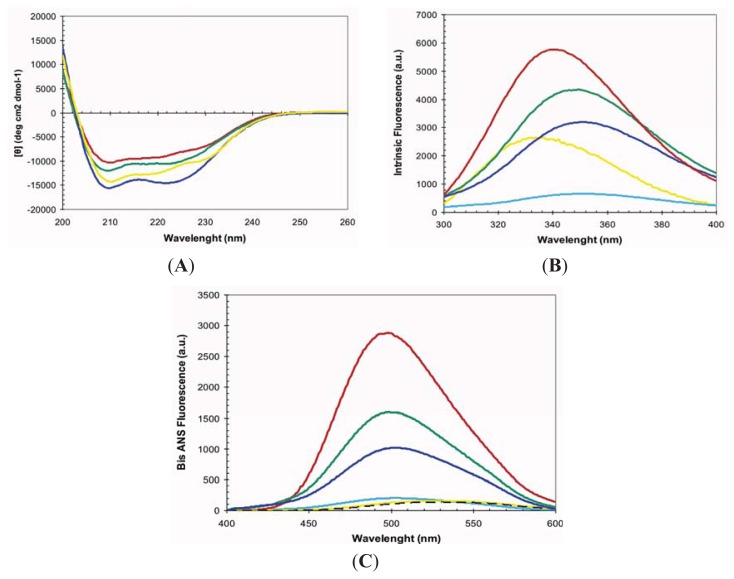
2,2,2-trifluoroethanol (TFE) dependence of URN1-FF molten globule (MG) conformational properties. Protein samples were prepared at 20 μM and pH 2.5 and immediately measured by (**A**) far-UV CD, (**B**) tryptophan intrinsic fluorescence and (**C**) 4,4′-Dianilino-1,1′-Binaphthyl-5,5′-Disulfonic Acid (bis-ANS) fluorescence at 25 °C. The fluorescence emission spectrum of bis-ANS in the absence of protein is represented as a dotted line. URN1-FF species were measured at 0% (red), 15% (green) and 25% (blue) of TFE (*v*/*v*). The spectra of the native protein at pH 5.7 are shown in yellow. In (**B**) and (**C**) the spectrum of heat denatured URN1-FF at 90 °C is shown in light blue.

At pH 2.5 the URN1-FF forms an MG state that retains most of the α-helical secondary structure content characteristic of the protein native state [27]. Accordingly, in these conditions, the far-UV CD spectrum indicates that most of the polypeptide chain adopts an α-helical conformation and the spectra is similar to that of the native protein at pH 5.7 (Figure 2A). The URN1-FF contains two buried Trp residues at positions 27 and 56 (Figure 1). The intrinsic fluorescence spectra of the acid induced URN1-FF MG displays higher intensity and is slightly red-shifted compared with that of the native state suggesting an opening of the globular structure with a concomitant exposure of hydrophobic clusters to the solvent at low pH (Figure 2B). This extent is confirmed by the observed increase in the fluorescence emission of the dye 4,4′-Dianilino-1,1′-Binaphthyl-5,5′-Disulfonic Acid (bis-ANS) and the blue-shift of its maximum in the presence of the protein at low pH, when compared with the spectrum of bis-ANS in the presence of the native protein (Figure 2C). Despite the fact that the acid induced URN1-FF MG does not exhibit any evidence of the population of β-sheet conformations, this state is aggregation competent [27], which contrasts with the native state, where the protein remains soluble for years. Two main reasons might account for the different solubility of the two states: (i) opening of the tertiary structure and associated exposition of previously hidden non-polar residues would promote the establishment of hydrophobic intermolecular contacts leading to the formation of aggregation nuclei; (ii) fluctuations of the helical secondary structure would result in the transient formation of disordered protein regions able to establish intermolecular backbone hydrogen bonds, leading to formation of β-sheet enriched oligomers. The first mechanism has been proposed to be involved in the aggregation of proteins belonging to different structural classes, whereas the second one has been proposed to apply for proteins enriched in α-helical structures such us myoglobin [28]. To decipher the contribution of these two effects to the aggregation of URN1-FF we investigated the conformational and aggregational properties of the protein in the presence of 15% and 25% TFE (*v*/*v*).

The far-UV CD of URN1-FF at pH 2.5 in the presence of both 15% and 25% TFE (*v*/*v*) correspond to that of an all α-helical protein (Figure 2A). Accordingly, analysis of the CD spectra with the K2D3 algorithm predicts a secondary structure content consisting of ≈95% α-helix, without any significant β-sheet component. Both spectra display higher ellipticity at 210 nm and 222 nm than that of the MG state at the same pH, although only 25% TFE (*v*/*v*) promotes higher ellipticity than that in the native state.

At pH 2.5, the presence of TFE induces an increase and a red shift in the intrinsic Trp fluorescence of URN1-FF (Figure 2B), indicating that the presence of the cosolvent likely promotes additional opening of the structure. However, the spectra are still significantly different from that of denatured URN1-FF, where a strongest shift and reduction in the fluorescence emission is observed, suggesting that in the presence of TFE the protein presents at least a residual compact structure. In agreement with the Trp spectral data, the presence of TFE induces a reduction in bis-ANS fluorescence (Figure 2C), suggesting the disruption of certain hydrophobic interactions still present in the MG state. However, this reduction in bis-ANS binding is moderate if we compare it with that of the denatured state where the bis-ANS fluorescence decreases dramatically and red shifts. Overall, the data suggest that the presence of TFE promotes a conformation with a well-defined α-helical secondary structure, as observed for other amyloidogenic peptides [29], but only rudimentary tertiary contacts and highly accessible hydrophobic side-chains. These properties would allow us to study if it is the presence of solvent exposed non-polar residues or alternatively the weakening of the secondary structure that is mainly responsible for the aggregation of the URN1-FF domain when incubated at acidic pH.

### 2.2. Effect of TFE on Amyloid Fibril Formation by the URN1-FF Domain

**Figure 3:**
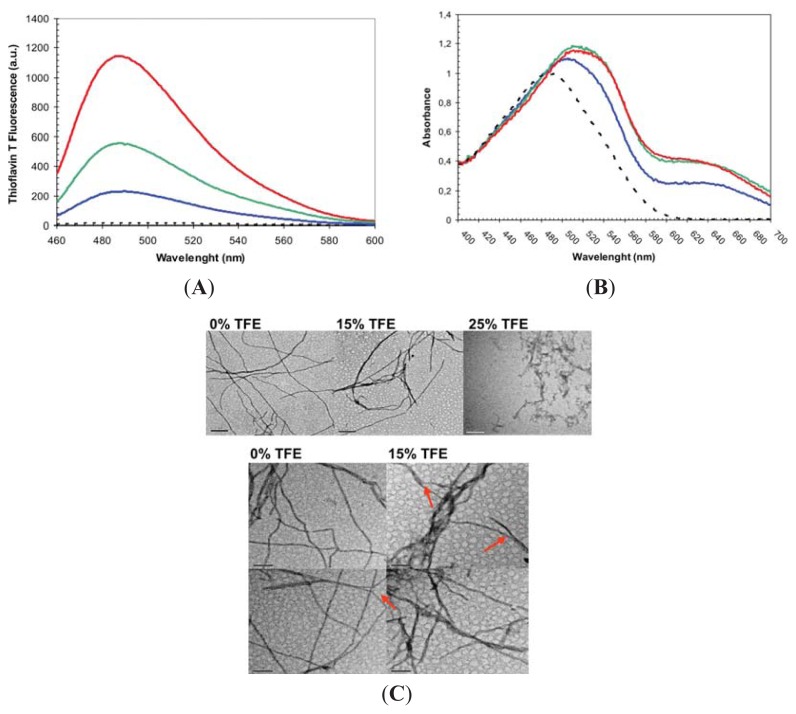
Amyloid-like properties of URN1-FF aggregates formed at different TFE concentrations. The protein domain was incubated at pH 2.5 and 100 μM for one week. (**A**) Fluorescence emission spectra of ThT (25 μM) in the absence (dotted line) and in the presence of 10 μM of protein aggregates; (**B**) Absorption spectra of CR (20 μM) in the absence (dotted line) and in the presence of 12 μM of URN1-FF aggregates, in (**A**) and (**B**) protein aggregates formed at 0%, 15% and 25% TFE (*v*/*v*) concentrations are represented in red, green and blue, respectively; (**C**) Representative TEM images of URN1-FF solutions incubated at the indicated TFE concentrations. The bar corresponds to 200 nm and 100 nm in upper and lower panels, respectively. The arrows indicate the assembly of thin fibrils to form ticker fibrillar structures.

The MG formed by URN1-FF at pH 2.5 is a stable species that remains apparently soluble for weeks at 20 μM concentration. However, it self-assembles into amyloid fibrils upon incubation at higher concentrations. We incubated the domain at 100 μM for 7 days at acid pH in the absence or the presence of 15% and 25% TFE (*v*/*v*) and analyzed the presence of amyloidogenic aggregates by monitoring the binding to Thioflavin-T (Th-T) of these protein solutions (Figure 3A). Despite all the samples promoting an increase in Th-T fluorescence emission indicative of the presence of amyloid-like protein aggregates, the presence of 15% and 25% TFE (*v*/*v*) reduced Th-T emission by 2.5 and 6 fold, respectively. We further explored the amyloid properties of the incubated protein solutions by monitoring the changes they induced in Congo Red absorbance spectrum. All the samples promoted the characteristic amyloid-induced red-shift and an increase in absorbance in the dye spectrum (Figure 3B). However, the binding to CR decreases in the presence of 25% (*v*/*v*) TFE. The presence and morphology of protein aggregates was further analyzed using Transmission Electron Microscopy (TEM) (Figure 3C).

Both in the absence and presence of 15% TFE (*v*/*v*) all the detected aggregates correspond to long fibrillar species. The fibrils in both solutions were long and unbranched and consist of linear thin and thick fibrils. Linear thin fibrils displayed a diameter of ~7.0 nm consistent with that of the amyloids formed by disease-linked polypeptides, which diameters in the 4–10 nm range [30]. Thick fibrils had a diameter of ~14 nm, which suggest that they likely result from the association of two thin fibrils and in fact the presence of these individual fibrils could be observed in some images. In the absence of TFE thin fibrils dominate the solution, whereas in the presence of 15% TFE thick fibrils become more abundant species. The presence of 25% (*v*/*v*) almost complete abrogated the presence of long fibrils and short protofibrilar assemblies become the main aggregated species.

**Figure 4:**
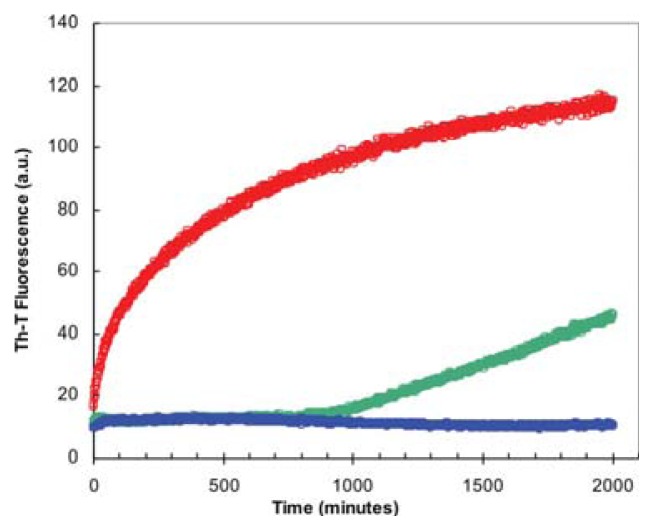
TFE dependence of URN1-FF aggregation kinetics. Change in Thioflavin-T (ThT) fluorescence (25 μM) during the aggregation of URN1-FF at 100 μM. Reactions at 0%, 15% and 25% TFE (*v*/*v*) concentrations are represented in red, green and blue, respectively.

We monitored if the presence of TFE affects the kinetics of URN1-FF amyloid fibril formation by following the increase in ThT fluorescence emission during 2000 min (Figure 4). The kinetics of amyloid fibril formation can be usually adjusted to a sigmoidal curve, reflecting the existence of a nucleation-dependent growth reaction. The aggregation of URN1-FF at acidic pH follows this kinetic scheme but exhibits a very short lag phase of only few minutes (Figure 4) suggesting that the formation of amyloid-like intermolecular interactions occurs rapidly in the aggregation process. The presence of 15% TFE (*v*/*v*) has a dramatic effect on the kinetics of URN1-FF amyloid formation extending the lag phase up to 15 h. Moreover, no evident aggregation could be observed during the time of the experiment in the presence of 25% TFE.

Overall, the presence of TFE at moderate concentrations has an inhibitory effect on the process of amyloid fibril formation by the URN1-FF domain at low pH. This effect contrasts with the pro-aggregational effect shown for TFE in the same concentration range for a wide range of proteins, indicating that the effect of this cosolvent is not generic and depends on the conformational properties of the target protein. The kinetic assays indicate that this fluoroalcohol acts at the early stages of the aggregation reaction making more difficult or impeding the structural transition from an initially monomeric and soluble form to aggregation-prone species. The conformational characterization of the protein in the absence and presence of the cosolvent suggests that this effect is exerted through a reinforcement of the global helical propensity of the protein. TFE is a solvent less polar than water and a weaker hydrogen bond competitor. Accordingly, it has been suggested that its ability to promote the formation of ordered aggregates depends on an strengthening of the interactions that stabilize intermolecular β-sheet structure as well as intramolecular β-turns; however, our data suggest that in the case of all α-helical proteins, such as URN1-FF, it also promotes a reinforcement of the backbone hydrogen bonds sustaining the helical structure, which competes with the formation of the intermolecular hydrogen bonds necessary to align the main chain in β-sheet enriched conformations. In contrast to what is observed in other protein models containing preformed β-strands in their native state, by favoring stronger interactions, TFE changes the balance factors that favor solubility relative to aggregation, since in α-helical proteins they result in the formation of native-like contacts, whereas for β-sheet containing proteins its effect is disruptive and promote non-native intermolecular binding. In URN1-FF, this over-stabilizing effect of the native secondary structure can overcome the disruption of the tertiary structure and thus the presence of hydrophobic residues exposed to solvent. In support of this view, it has been shown that mutations in proteins that stabilize α-helical structure can slow down the process of aggregation from their denatured states [31].

### 2.3. Seeding Properties of URN1-FF Domain Aggregates

**Figure 5:**
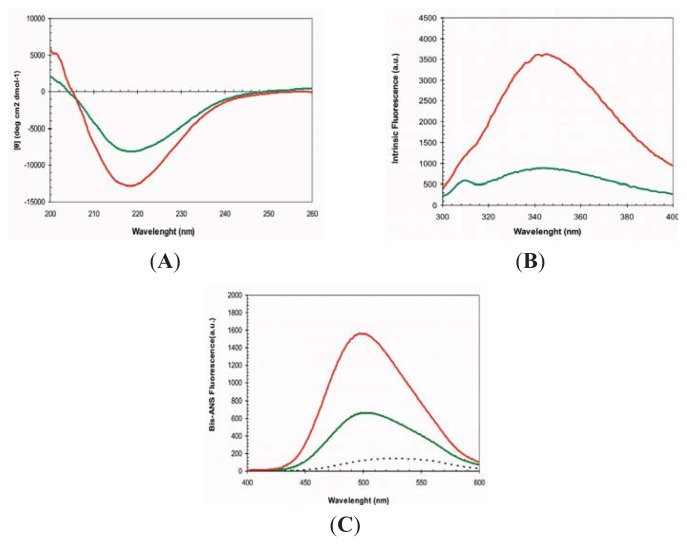
TFE dependence of the conformational properties of URN1-FF fibrils. The protein domain was incubated at pH 2.5 and 100 μM for at least 21 days. (**A**) Far-UV CD spectra using a final concentration of 20 μM; (**B**) Tryptophan intrinsic fluorescence; (**C**) Fluorescence emission spectra of bis-ANS (25 μM) collected in the absence (dotted line) and in the presence of fibrils (10 μM). In all the cases, f0% fibrils in red and f15% fibrils in green.

We monitored the conformational properties of the amyloid structures formed in the absence and presence of 15% of TFE in order to analyze if, as with the soluble protein, they exhibit differences in these two conditions. To this aim we used protein solutions incubated for at least 21 days, since quantification of the amount of aggregated protein by sample fractionation using sedimentation at 100,000g for 1 h indicated that >95% of the total protein was located in the insoluble fraction both in the absence and presence of the cosolvent. The fibrils formed in the absence and presence of 15% TFE (*v*/*v*) will be referred to as f0% and f15% fibrils, respectively. The far-UV CD spectra of these protein solutions indicate that in both cases a transition from the original helical structure towards a β-sheet enriched conformation has occurred, since both spectra present a characteristic minimum at ~217 nm, without any remaining α-helical component (Figure 5A). We also monitored the Trp intrinsic fluorescence emission in both fibrilar states. In contrast to what happens in the soluble states, the f15% fibrils display lower fluorescence emission, suggesting that likely their hydrophobic residues, including Trp, are more buried (Figure 5B). This is also consistent with their lower binding to bis-ANS, relative to the f0% fibrils (Figure 5C). Although speculative, it is tempting to propose that these conformational differences might be related to the different persistence of thick fibrils in the two solutions, in such a way that the association of two thin fibrils might occur through hydrophobic residues that become therefore protected from the solvent.

**Figure 6:**
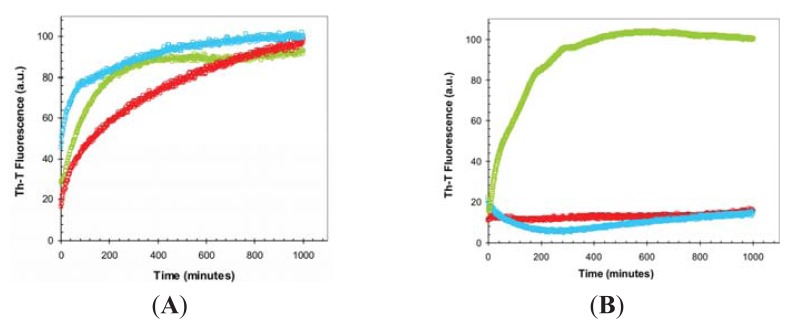
TFE dependence of the self-propagation of URN1-FF amyloid fibrils originating from various concentrations of TFE. Change in ThT fluorescence (25 μM) during the aggregation of URN1-FF at 100 μM. 10% of preformed fibrils were used for seeding and cross-seeding assays. (**A**) Aggregation reactions in the absence of TFE and (**B**) the presence of 15% TFE (*v*/*v*). Unseeded reactions are shown in red, reactions seeded with f0% and f15% fibrils are shown in blue and green, respectively.

The nucleation step of the amyloid assembly is shortened in the presence of preformed amyloid fibrils of the same protein that can act as seeds for the polymerization reaction. This reaction is not only sensible to the protein sequence but also to the protein conformation and it has been shown for different models that fibrils of the same protein formed under different conditions might have differential seeding properties. In contrast, in specific cases, proteins of dissimilar sequences might cross-seed [32]. We tested if this is the case for f0% and f15% URN1-FF fibrils. First, we addressed if the amyloid aggregation reaction of this domain at low pH and aqueous solution can in fact be seeded be the fibrils preformed in the same conditions. To this aim we added 10% of sonicated preformed fibrils at the beginning of the polymerization reaction. As shown in Figure 6, in the presence of these seeds, the reaction is accelerated significantly at early stages, indicating that they exert the nucleating effect characteristic of amyloids. Next, we tested if preformed f15% fibrils were able to seed the reaction when added to an URN1-FF solution devoid of the cosolvent. Figure 6A indicates that this is the case, and despite the accelerating effect being lower than that of the fibrils formed in the absence it is still very significant.

Thus, despite the different spectroscopic features of the two types of aggregates it is likely that they share some common molecular details that allow them to cross-seed. Next we addressed if f0% and f15% URN1-FF preformed fibrils would be able to short the long lag phase of the aggregation reaction when it occurs in the presence of the 15% TFE (*v*/*v*) (Figure 6B). The f15% fibrils efficiently seeded the aggregation of the soluble protein in the presence of the cosolvent. Surprisingly, the f0% fibrils did not exert any accelerating effect on the aggregation reaction in this condition. This indicates that presence of TFE not only acts by modulating the formation of the initial nucleus but also induces structural diversity in URN1-FF fibrils. Our results are consistent with recent data obtained for barstar, in which the protofibrils formed in the presence and absence of trifluorethanol were shown to differ not only in the orientation and number of β-sheet motifs but also in the number of protein residues involved in the maintenance of the protofibrillar structure [33].

**Figure 7:**
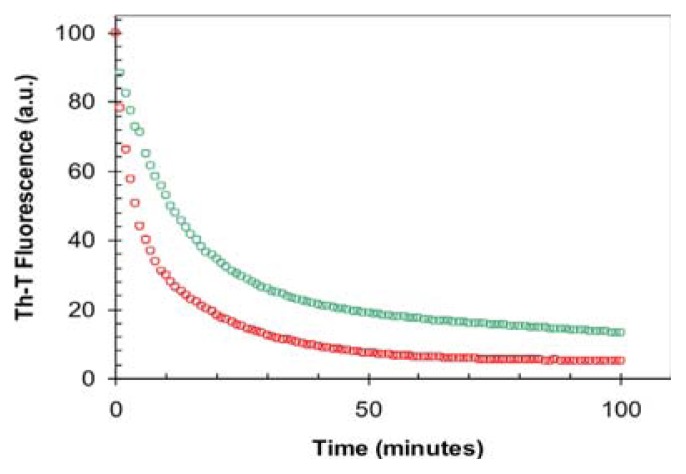
Depolymerization of URN1-FF fibrils in the presence of 15% TFE. Change in ThT fluorescence (25 μM) after 5-fold dilution of URN1-FF f0% (red) and f15% (green) fibrils in 15% TFE, shown in arbitrary units (a.u.).

The lack of seeding capability of f0% fibrils when the reaction occurs in the presence of TFE indicates that they differ in conformation to f15% fibrils; however, since both f0% and f15% fibrils are able to seed the aggregation reaction in aqueous solution, it is difficult to argue that they will notshare that f0% fibrils depolymerizate fast in the presence of TFE, which would preclude them to act as effective seeds in this condition. In support of this view, when f0% fibrils were diluted in 15% TFE (*v*/*v*) the sequential/structural determinants responsible for amyloid propagation. Yet another possibility is the Th-T fluorescence decreased rapidly towards the control intensity obtained without fibrils, indicative of a high rate of depolymerization in the presence of the cosolvent (Figure 7). Despite the fact that f15% fibrils also exhibit a decay in Th-T binding upon dilution, the resulting reaction is 6-fold slower and the final Th-T fluorescence stronger, indicating that a higher population of f15% fibrils remained at equilibrium. Similar to our results, also the fibrils formed by the amyloidogenic β2-microglobulin protein under different TFE concentrations differ in their conformational properties, in such a way that the fibrils with slower depolymerization reactions are those more formed at higher concentrations of TFE, exhibiting at the same time the slower aggregation kinetics [34]. This apparent paradox can be solved if one considers α-helix and cross-β sheet as two sides of the same coin, both structures being stabilized by extended networks of hydrogen bonds, the formation of which is facilitated in the presence of TFE. As a result, in proteins like the URN1-FF domain, displaying intrinsic α-helical propensity, the presence of TFE would result in a high energy barrier of fibrillation. This barrier would be much lower in proteins containing regions with low helical propensity, such as β-strands, explaining why, in those cases, TFE act at moderate concentrations as a pro-aggregational compound.

The toxicity of amyloid fibrils is related to their conformational properties. It has been shown for different and unrelated proteins that the binding to ANS-like dyes correlates with the toxicity of amyloid species, suggesting that the exposure of hydrophobic regions is a critical characteristic of these pathogenic assemblies. Although the aggregation of URN1-FF is not associated to any known disease, the striking different binding of the fibrils formed in the absence and presence of TFE to bis-ANS suggest that the polarity of the microenvironment might be an important determinant of the toxicity of the resulting fibrils, an effect that could be relevant inside the cell where aggregation might occur in the cytosol but also at the membrane surface, which lipid molecules resemble in their chemical properties to TFE, a cosolvent usually employed as a membrane mimic.

## 3. Experimental Section

### 3.1. Protein Expression and Purification

Competent *E. coli* BL21(DE3) cells were transformed with a pETM-30 plasmid encoding the URN1-FF domain that corresponds to residues 212–266 of yeast URN1; it was cloned as an *N*-terminal fusion protein with a His tag followed by GST and a TEV protease cleavage site. Transformed cells were incubated in Luria Bertani medium overnight at 37 °C and then diluted to 1/100 (*v*/*v*). After growing to 0.6 optical density they were induced with 1 mM IPTG overnight at 25 °C. A His-tag column was used to isolate the URN1-FF fusion. The GST protein was removed by TEV cleavage and a final gel filtration on a HiLoad™ Superdex™ 75 prepgrade column (Amersham Pharmacia Biotech AB, Uppsala, Sweden). The protein was dialyzed against water and lyophilized. Protein concentration was determined by UV absorption using a ɛ value of 1.948 mg^−1^·mL·cm^−1^.

### 3.2. Protein Preparation for URN1-FF Conformational Assays

Lyophilized URN1-FF protein was prepared at 20 μM in 50 mM glycine at pH 2.5 and different amounts of TFE were added. Protein solution was filtered through a 0.22 μm filter and immediately analyzed at 25 °C.

### 3.3. Protein Preparation for URN1-FF Aggregation Assays

Lyophilized URN1-FF protein was dissolved at 100 μM in 100 mM glycine at pH 2.5 in the presence or absence of TFE and filtered through a 0.22 μm filter. The samples were incubated under agitation at 400 rpm and 37 °C.

### 3.4. Circular Dichroism and Intrinsic Tryptophan Fluorescence

Monomeric and aggregated URN1-FF species were dissolved at 20 μM and measured immediately. Far-UV CD spectra were measured in a Jasco-710 spectropolarimeter (Jasco Corporation, Hachioji, Japan) thermostated at 25 °C. Spectra were recorded from 260 to 200 nm, at 0.2 nm intervals, 1 nm bandwidth, and a scan speed of 200 nm/min. For each spectrum were averaged twenty accumulations. Tryptophan intrinsic fluorescence was measured at different temperatures in a Cary-100 Varian spectrofluorometer (Varian, Inc., Palo Alto, CA, USA) using an excitation wavelength of 280 nm and recording the emission from 300 to 400 nm. Three averaged spectra were acquired and slit widths were typically 5 nm for excitation and emission.

### 3.5. bis-ANS Binding Assay

Aggregated samples were diluted at 10 μM in phosphate buffer pH 7.5 containing 25 μM of bis-ANS. To study soluble URN1-FF species, samples were prepared at 20 μM containing 25 μM of bis-ANS and analyzed immediately. The excitation wavelength was 370 nm and the emission spectra was recorded between 400 and 600 nm, using excitation and emission slit widths of 5 nm. Three spectra were accumulated after 5 min of equilibration at different temperatures in a CARY-100 Varian Spectrophotometer (Varian, Inc., Palo Alto, CA, USA).

### 3.6. Binding to ThT

Aggregated URN1-FF was diluted to 10 μM in phosphate buffer at pH 7.5 in the presence of 25 μM of ThT. The sample was excited at 440 nm and fluorescence emission was acquired between 460 and 600 nm, using excitation and emission slit widths of 5 nm. Each trace was the average of 3 accumulated spectra at 25 °C in a CARY-100 Varian spectrophotometer (Varian, Inc., Palo Alto, CA, USA).

### 3.7. Binding to Congo Red

Thirty microliters of aggregated URN1-FF were added to 220 μL of CR (20 μM) in 5 mM phosphate, 150 mM NaCl pH 7.4 buffer at 25 °C. After 5 min of equilibration, optical absorption spectra were recorded from 400 to 700 nm and accumulated for 3 times with a Jasco V-630 spectrophotometer (Jasco Corporation, Hachioji, Japan). Solutions containing only protein and only CR were analyzed to eliminate the protein scattering and dye contribution to the spectra.

### 3.8. Electron Microscopy

The analysis was performed using a HITACHI H-7000 transmission electron microscope (Hitachi, Tokyo, Japan) operating at an accelerating voltage of 75 kV. Ten microliters of incubated samples were diluted tenfold with water and were placed on carbon-coated copper grids. After 5 min the grids were washed with distillated water and stained with 2% (*w*/*v*) uranyl acetate for 1 min.

### 3.9. Aggregation Kinetics and Seeding Assays

URN1-FF protein was prepared at 100 μM in 100 mM glycine at pH 2.5 in the presence of 25 μM of ThT and 0%, 15% and 25% TFE (*v*/*v*). Immediately after equilibrating the sample at 37 °C during 5 min, ThT intrinsic fluorescence was measured every 2 min. The sample was excited at 440 nm and emission was recorded at 475 nm for ThT. Slitwidths of 5 nm and 10 nm were used for excitation and emission respectively in a CARY-100 Varian spectrophotometer (Varian, Inc., Palo Alto, CA, USA). For seeding assays, 10% (*v*/*v*) of preformed fibrils formed under different conditions were added to soluble URN1-FF at 100 μM in the absence or presence of TFE, immediately before incubation; the kinetics were followed as described above.

### 3.10. Depolymerization Assays

Ten percent (*v*/*v*) of preformed fibrils formed under different conditions were diluted 5-fold in 100 mM glycine at pH 2.5 containing 15% of TFE (*v*/*v*). The sample was excited at 440 nm and emission was recorded at 475 nm for ThT. Slitwidths of 5 nm and 10 nm were used for excitation and emission respectively in a CARY-100 Varian spectrophotometer (Varian, Inc., Palo Alto, CA, USA). ThT intrinsic fluorescence was measured every minute.

## 4. Conclusions

Experimental characterization of TFE-induced changes in the FF domain aggregation process suggests that microenviroments affecting the competition between native-like and amyloidogenic contacts might influence not only the transition between the soluble and fibrillar states, but also the conformational properties of the aggregated assemblies, and thus potentially their cytotoxicity. In this context, it is feasible that chemical compounds that selectively stabilize native α-helices in proteins will increase the energy barrier of fibrillation and therefore can be used to fight amyloid formation at a molecular level [35].

## Abbreviations

bis-ANS: 4,4′-Dianilino-1,1′-Binaphthyl-5,5′-Disulfonic Acid
CD: circular dichroism
CR: Congo red
MG: molten globule state
TFE: 2,2,2-trifluoroethanol
Th-T: Thioflavin-T
URN1-FF: FF domain of the URN1 splicing factor
a.u.: arbitrary units
